# The Anticancer Activity of Sea Buckthorn [*Elaeagnus rhamnoides* (L.) A. Nelson]

**DOI:** 10.3389/fphar.2018.00232

**Published:** 2018-03-15

**Authors:** Beata Olas, Bartosz Skalski, Karolina Ulanowska

**Affiliations:** Department of General Biochemistry, Faculty of Biology and Environmental Protection, University of Lodz, Lodz, Poland

**Keywords:** *Elaeagnus rhamnoides* (L.) A. Nelson, sea buckthorn, cancer, antioxidant, phenolics, berry

## Abstract

Various parts of sea buckthorn [*Elaeagnus rhamnoides* (L.) A. Nelson], particularly the berries, known also as seaberries, or Siberian pineapples, are characterized by a unique composition of bioactive compounds: phenolic compounds, vitamins (especially vitamin C), unsaturated fatty acids, and phytosterols such as beta-sitosterol. These berries, together with the juices, jams, and oils made from them, have a range of beneficial antioxidant, anti-inflammatory, and anticancer effects. This short review discusses whether sea buckthorn may represent a “golden mean” for the treatment of cancers: It has anti-proliferation properties and can induce apoptosis and stimulate the immune system, and sea buckthorn oil counteracts many side effects of chemotherapy by restoring kidney and liver function, increasing appetite, and keeping patients in general good health. Although the anticancer activity of sea buckthorn has been confirmed by many *in vitro* and animal *in vivo* studies, the treatment and prophylactic doses for humans are unknown. Therefore, greater attention should be paid to the development of well-controlled and high-quality clinical experiments in this area.

## Introduction

Sea buckthorn [*Elaeagnus rhamnoides* (L.) A. Nelson; *Hippophae rhamnoides L*. (old name)] is a member of the *Elaeagnaceae*. It is currently cultivated on a production scale, primarily in Russia and China, and in a growing number of varieties around the world (i.e., Finland, Germany, and Estonia).

Both *in vitro* and human and animal *in vivo* studies on sea buckthorn have found a range of bioactive chemicals in its leaves, roots, seeds, and berries, known as seaberry, or Siberian pineapple, as well as the oil extracted from them; these compounds exhibit a wide range of anti-inflammatory, anticancer, antioxidant, and anti-atherosclerotic activities (Zeb, 2006; Basu et al., 2007; Kumar et al., 2011; Suryakumar and Gupta, 2011; Xu et al., 2011; Christaki, 2012; Teleszko et al., 2015; Olas, 2016; Ulanowska et al., in press). Several trace elements and vitamins (especially A, C, and E), lipids, carotenoids, amino acids, unsaturated fatty acids, and phenolic compounds that are found in the berries are presented in Table 1 (Olas, 2016; Gradt et al., 2017; Ulanowska et al., in press). Their concentration in the berries depends on the climate, size, maturity of the plant, and the procedure used to process and store the plant material (Fatima et al., 2012; Malinowska and Olas, 2016). Gao et al. (2000) report changes in antioxidant properties, as well as other types of biological activity, in sea buckthorn berries during maturation, which were strongly correlated with the content of total phenolic compounds and ascorbic acid. Moreover, the antioxidant activity of the lipophilic extract increased significantly and corresponded to the increase in total carotenoid content.

**Table 1:** The chemical composition of individual parts of the sea buckthorn (44; modified).

**Part of sea buckthorn**	**Chemical composition**
Fruits (berries)	Vitamins (C, E, B, K_1_, D, A, folic acid) Macro and trace elements (potassium, magnesium, calcium, iron, sodium, manganese, zinc, copper, nickel) Carotenoids Phenolic compounds Lipids Amino acids Organic acids Proteins Sugars Pectins
Leaves	Vitamins (E, folic acid) Calcium, magnesium, potassium Carotenoids Phenolic compounds Amino acids Chlorophyll Proteins Pectins
Seeds	Carotenoids Phenolic compounds Lipids Proteins
Roots	Carotenoids Phenolic compounds Lipids Proteins
Bark	Phenolic compounds

A wealth of healthy ingredients are found not only in the raw fruits, but also in a variety of preparations such as jams, juices, marmalades, or tinctures. Sea buckthorn berries can be also used to make pies and liquors (Li and Hu, 2015). Hu (2005) reports that sea buckthorn seed can be used to make oil and the leaves can used to make tea. While teas made from the seeds have laxative properties and help weight loss, infusions of the leaves have antidiarrheal properties; in addition, fruit teas strengthen the immune system, and show activity against skin diseases (Frohne, 2010; Sarwa, 2001).

The positive and unique properties of sea buckthorn have been known since at least the VII Century BC (Suryakumar and Gupta, 2011; Li and Hu, 2015). The plant was used not only in natural medicine, but also veterinary medicine as a means of relieving helminthiasis in horses and providing them more mass and a beautiful, shiny coat. Currently, its products are used in many industries, especially the pharmaceutical, cosmetic, and food industries, but also as a decorative element, as firewood, or even as a tool for the rehabilitation of degraded areas. According to historical records, sea buckthorn was first used as a drug in China, and in more modern times, the plant was formally listed in the Chinese Pharmacopoeia in 1977 (The State of Pharmacopoeia Commission of PR China, 1977).

Modern studies have shown that the parts of sea buckthorn can serve as natural remedies for cardiovascular diseases, as well as diseases of the skin, liver, and stomach. The therapeutic potential of its bioactive compounds is demonstrated in Table 2. This review article summarizes the current knowledge concerning the different organs of sea buckthorn, and discusses whether they may represent a “golden mean” for the treatment of cancer. It is important to note that the source information for this paper is derived not only from *in vitro* models, but also *in vivo* models.

**Table 2:** Sea buckthorn bioactive compounds and their therapeutic effects (44; modified).

**Bioactive compound**	**Therapeutic effect**
Tocopherol	Antioxidant Analgesic action Protection against degenerative changes, thrombosis, and muscle cramps
Carotenoids	Antioxidant Involved in the synthesis of collagen Protection and restoration of the mucous membranes and epithelia Enhancing the immune system
Phytosterols	Anti-atherosclerotic action, anti-inflammatory and antibacterial properties The prophylaxis and treatment of hypercholesterolemia-induced cardiovascular disorders by lowering serum cholesterol concentrations Reducing the risk of stomach ulcers
Unsaturated fatty acids	Protecting against cerebrovascular and cardiovascular disorders Stimulating the immune system Promoting cognitive function and bone health. A positive effect on such neurological disorders as depression, schizophrenia, and Alzheimer's disease
Organic acids	Acceleration of wound healing Protecting against cerebrovascular and cardiovascular disorders
Vitamin C	Antioxidant Involved in the synthesis of collagen Maintaining correct cell membrane integrity
Vitamin K	Prevention of bleeding Reducing the risk of stomach ulcers Assisting the reconstruction of skin damage
Phenolic compounds	Antioxidant Reducing the risk of cardiovascular disease Involved in regulating heart rhythm Prevention of tumors Alleviating the symptoms of aging

## Anticancer activity of sea buckthorn

A number of phytopharmaceuticals, particularly such phenolic compounds as proanthocyanidins, curcumin, and resveratrol, have been found to offer significant benefits in cancer chemoprevention (Barrett, 1993; Bagchi and Preuss, 2004; Bagchi et al., 2014; Shanmugam et al., 2015; Ko et al., 2017) and radiotherapy (Cetin et al., 2008). It is well-documented that higher dietary intakes of phenolic compounds, especially procyanidins and flavonoids are associated with a lower risk of cancer (Barrett, 1993; Bagchi and Preuss, 2004; Duthie et al., 2006; Zafra-Stone et al., 2007; Cetin et al., 2008; Seeram, 2008; Bagchi et al., 2014; Chen et al., 2014; Wang et al., 2014; Giampieri et al., 2016; Kristo et al., 2016). Sea buckthorn possesses a wide range of biological and pharmacological activities, including anticancer properties. Although the molecular mechanisms underlying them remain unclear, these compounds are known to be present in different organs and their products, especially in the juice and oil (Xu et al., 2011). The antitumor activity of sea buckthorn can be attributed to antioxidant compounds, particularly phenolic compounds such as flavonoids, including kaempferol, quercetin, and isorhamnetin; these protect cells from oxidative damage that can lead to genetic mutation and to cancer (Christaki, 2012).

### *in vitro* studies

Various *in vitro* studies have demonstrated that sea buckthorn has anticancer activity. For example, Zhang et al. (2005) investigated changes in the expression of apoptosis-related genes in the human breast carcinoma cell line Bcap-37 induced by flavonoids from sea buckthorn seed. Their bioinformatics analysis found that the expression of 32 analyzed genes, including CTNNB1, IGFBP4, GADD34, and caspase 3, associated with the apoptosis of Bcap-37 cells, was influenced by flavonoid treatment.

Teng et al. (2006) found that isorhamnetin (3′-methoxy-3,4′5,7-tetra hydroxyl flavone; a flavonoid isolated from sea buckthorn) has cytotoxic effects against human hepatocellular carcinoma cells (BEL-7402), with an IC_50_ of about 75 μg/ml after 72-h treatment. Li et al. (2015) also found isorhamnetin to have anti-proliferation effects on lung cancer cells *in vitro* when applied at concentrations ranging from 10 to 320 μg/ml, and *in vivo* in C57BL/6 mice when administrated orally (50 mg/kg/d) for 7 days. The authors suggest that the mechanism of isorhamnetin action may involve the apoptosis of cells induced by the down-regulation of oncogenes and up-regulation of apoptotic genes. Other observations showed that isorhamnetin suppresses the proliferation of cells from the human colorectal cancer cell lines (HT-29, HCT 116, and SW480), induces cell cycle arrest at the G2/M phase, and suppresses cell proliferation by inhibiting the PI3K-Akt-mTOR pathway. In addition, isorhamnetin reduced the phosphorylation levels of Akt (Ser473), phosph-p70S6 kinase, and phosph-4E-BP1 (t37/46) protein, and enhanced the expression of cyclin B1 protein at concentrations of 20 and 40 μM (Li et al., 2014).

In a study on MDA-MB-231 human breast cancer cells, Wang et al. (2014) noted sea buckthorn procyanidins isolated from the seeds to have inhibitory effects on fatty acid synthase (FAS): a key enzyme for *de novo* long-chain fatty acid biosynthesis, high levels of which are found in cancer cells. This inhibition was dose-dependent at concentrations ranging from 0 to 0.14 μg/ml. A concentration of 0.087 μg/ml inhibited 50% of FAS activity. Moreover, cell growth was suppressed by treatment with sea buckthorn procyanidins at concentrations between 10 and 60 μg/ml. In addition, the tested procyanidins were found to induce cell apoptosis in a dose-dependent manner. The authors suggest that these procyanidins can induce MDA-MB-231 cell apoptosis by inhibiting intracellular FAS activity.

Olsson et al. (2004) compared the effect of 10 different extracts of fruits and berries, including sea buckthorn berries, on the proliferation of HT29 semi-colon cancer cells and MCF-7 breast cancer cells. They observed that sea buckthorn had the highest inhibition effect for the proliferation of HT29 and MCF-7 cells at its two highest administered concentrations (0.25 and 0.5%). The authors suggest that the inhibition of cancer cell proliferation was correlated with concentrations of carotenoids and vitamin C. Moreover, they propose the presence of a synergistic action between carotenoids, vitamin C, and anthocyanins. In addition, McDougall et al. (2008) note that sea buckthorn berry extract possessed slightly antiproliferative effects against cervical and a semi-colon cancer cells grown *in vitro*.

Boivin et al. (2007) determined the antiproliferative activity of the juices of 13 types of berries, including sea buckthorn, at concentrations of 10–50 μg/ml against five cancer cell lines *in vitro*: AGS—stomach adenocarcinoma, ACF-7—mammary gland adenocarcinoma, PC-3—prostatic adenocarcinoma, Caco-2—colorectal adenocarcinoma, and MDA-MB-231—mammary gland adenocarcinoma. It was found that sea buckthorn berry juice, like blackberry and black chokeberry juices, had anti-proliferative properties. However, no correlation was found between the anti-proliferative properties of the berry juices and their antioxidant capacity, and the inhibition of cancer cell proliferation by the juices did not involve caspase-dependent apoptosis. Despite this, suppression of tumor necrosis factor (TNF)-induced activation of nuclear factor kappa-light-chain-enhancer of activated B cells (NFκB) was observed.

Recently, Guo et al. (2017) studied the phytochemical composition of the berries of four different subspecies of sea buckthorn, as well as their antioxidant and antiproliferative properties against HepG2 human liver cancer cells *in vitro*: *H. rhamnoides* L. subsp. *sinensis* (Sinensis), *H. rhamnoides* L. subsp. *yunnanensis* (Yunnanensis), *H. rhamnoides* L. subsp. *mongolica* (Mongolica), and *H. rhamnoides* L. subsp. *turkestanica* (Turkestanica). Of these subspecies, *H. rhamnoides* L. subsp. *sinensis* demonstrated the highest total phenolic content [about 39 mg gallic acid (GA) equiv./g dry weight] and corresponding total antioxidant activity, while the greatest cellular antioxidant and antiproliferative properties were observed in *H. rhamnoides* L. subsp. *yunnanensis*. These properties were attributed to the action of phenolic acids and flavonoid aglycones.

Zhamanbaeva et al. (2014) studied the effects of ethanol extract from sea buckthorn leaves on the growth and differentiation of human acute myeloid leukemia cells (KG-1a, HL60, and U937). Although a plant extract was found to inhibit cell growth according to cell strain and extract dose, the study does not identify the chemical content of the tested extract. They used three concentrations of the extract: 25, 50, and 100 μg/ml. The findings suggest that the antiproliferative effect of sea buckthorn extract on acute myeloid leukemia cells was partially determined by activation of the S phase checkpoint, which probably led to deceleration of the cell cycle and induction of apoptosis.

Elsewhere, Zhamanbayeva et al. (2016) studied the antiproliferative and differentiation-enhancing activity of various plant extracts (10–100 μg/ml), including water-ethanol extract from leaves of sea buckthorn: it was found to have a total polyphenol content of approximately 46 mg GA equivalent/g dried extract, total flavonoid content of approximately 23 mg quercetin equivalent/g dried extract. The authors observed that the tested extracts, including sea buckthorn extract, reduced the growth and viability of acute myeloid leukemia cells; in addition, at non-cytotoxic doses, they also potentiated cell differentiation induced by a low concentration of 1α,25-dihydroxyvitamin D_3_, in a manner dependent on cell type. Moreover, the tested extracts strongly inhibited microsomal lipid peroxidation and protected normal erythrocytes against hypo-osmotic shock.

A recent study by Kim et al. (2017) proposes that sea buckthorn leaf extract, containing about 70 mg/g total phenolic compounds and about 460 μg/g catechin, may inhibit the rapid proliferation of rat C6 glioma cells when administered at 0.62, 6.2, and 62 μg/ml, probably by inducing the early events of apoptosis. The authors also suggest that the reduction of C6 glioma cell proliferation and viability following administration of the plant extract was accompanied by a decrease in the production of reactive oxygen species, which are critical for the proliferation of tumor cells. Moreover, sea buckthorn not only upregulated the expression of the pro-apoptotic protein Bcl-2-associated X (Bax), but also promoted its localization in the nucleus.

Various studies report that sea buckthorn oil also possesses anti-tumor properties. This oil can be incorporated in capsules, gelatin, and oral liquids (Yang and Kallio, 2002). Moreover, toxicity studies report no adverse effects in subjects administered with sea buckthorn oil (Upadhyay et al., 2009). Kumar et al. (2011) indicate that sea buckthorn oil plays an important role in cancer therapy, including chemotherapy and radiotherapy, and that taking sea buckthorn oil may help counteract many side effects or treatment, restore kidney and liver function, increase appetite, and generally keep patients in good health. Wang et al. (1989) observed that seed oil retarded tumor growth by 3–50%. Zhang et al. (Zhang, 1989) demonstrated that injection of seed oil (1.59 g/kg body weight) significantly inhibited the growth rate of transplanted melanoma (B_16_) and sarcoma (S_180_) tumors in mice. Wu et al. (1989) attribute the protective effect of sea buckthorn seed oil against cervical cancer to the presence of vitamins A and E. Finally, Sun et al. (2003) note that flavonoids from oil extracted from sea buckthorn seeds exert an inhibitory action on the liver cancer cell line BEL-7402 by inducing apoptosis.

The seeds and berry pulp of sea buckthorn contains various other bioactive compounds, including unsaturated fatty acids and phytosterols. It is known that unsaturated fatty acids have a multidirectional influence on human health, for example, by stimulating the immune system. In addition, phytosterols have anticancer properties (Sajfratova et al., 2010; Dulf, 2012). More details about the composition and beneficial health aspects of sea buckthorn oil are given by Olas (2018). The effect of sea buckthorn on cancer cells in different *in vitro* models is described in Table 3.

**Table 3:** The effect of sea buckthorn on cancer cells in *in vitro* models.

**Extract/chemical compound or other form obtained from sea buckthorn**	**Cancer cells**	**Concentration**	**Effect**	**Reference**
**EXTRACT OR OTHER FORM OBTAINED FROM SEA BUCKTHORN**				
Berry juice	Cell lines of breast cancer, prostate, stomach, and a semi-colon	10–50 μl/ml of medium	Inhibition of tumor cell proliferation of all tested lines	Boivin et al., 2007
Extract from berries in different solvents	Cancer cells of a semi-colon and liver	<0.1–2% (v/v) of medium	Inhibition of tumor cell proliferation of all tested lines; extract with ethyl acetate also caused apoptosis of these cells	Grey et al., 2010
Ethanol-water extract from berries	Cancer cells of the breast and a semi-colon	0.025–0.5% the dry weight in medium	Inhibition of tumor cell proliferation of all tested lines	Olsson et al., 2004
Ethanol-water extract from berries	Acute myeloid leukemia cells	10–100 μg/ml	Anti-proliferative action	Zhamanbayeva et al., 2016
Ethanol extract from berries	Acute myeloid leukemia cells	25, 50, and 100 μg/ml	Anti-proliferative action	Zhamanbaeva et al., 2014
Leaf extract	C6 glioma cells	0.62, 6.2, and 62 μg/ml	Anti-proliferative action	Kim et al., 2017
Isorhamnetin isolated from berries of sea buckthorn	Cancer cells of the liver	25–300 μg/ml of medium (IC_50_ = 75 μg/ml)	Cytotoxicity against cancer cells-a decrease in their vitality, fragmentation and chromatin condensation	Teng et al., 2006
Isorhamnetin isolated from berries of sea buckthorn	Lung cancer cells	10–320 μg/ml	Anti-proliferative action	Li et al., 2015
Isorhamnetin isolated from berries of sea buckthorn	Colorectal cancer cells	20 and 40 μM	Anti-proliferative action	Li et al., 2014
Procyanidins isolated from seeds	Breast cancer cells	10–60 μg/ml	Inducing apoptosis	Wang et al., 2014

### *in vivo* studies

Sea buckthorn has been found to have anticancer properties in both *in vitro* and *in vivo* studies on animal models. A study of the chemopreventive action of sea buckthorn fruits by Padmavathi et al. (2005) found them to inhibit dimethylobenzenoantracen-induced skin papillomagenesis in mice. The authors suggest that inhibition of carcinogenesis may be attributed to the concomitant induction of phase II enzymes, i.e., glutathione S-transferase, glutathione peroxidase, catalase, superoxide dismutase, and glutathione reductase in mouse liver. Moreover, the authors also suggest that the anticancer action of sea buckthorn fruits may be based on its enhancement of the DNA-binding activity of interferon regulatory factor-1 (IRF-1), a known antioncogenic transcription factor causing growth suppression and apoptosis.

Nersesyan and Muradyan (2004) report that sea buckthorn juice protects mice against the genotoxic action of cisplatin: a well-known anticancer drug which also is very toxic to normal cells. Sea buckthorn juice (300 ml) prepared *ex tempore* was given to mice by gavage for periods of 5 or 10 days. 3 h after the last gavage, mice received cisplatin at concentrations of 1.2 or 2.4 mg/kg.

Yasukawa et al. (2009) found 70% ethanol extract of sea buckthorn branches (1 mg of plant extract/mouse) to have antitumor properties in an *in vivo* two-stage carcinogenesis test with two groups of 15 mice; 7,12-dimethylbenz[a]anthracene as an indicator, and 12-*O*-tetracecanoyl-phorbol-13-acetate as a promotor. Of the three phenolic compounds (catechin, gallocatechin, and epigallocatechin) and the triterpenoid ursolic acid isolated from the extract, epigallocatechin, and ursolic acid were found to be the most active.

Wang et al. (2015) found that not only the phenolic compounds or phenolic extracts/fractions of sea buckthorn have anticancer properties: HRWP-A, a water-soluble homogenous polysaccharide with repeating units of (1 → 4)-β-D-galactopyranosyluronic residues, of which 85.2% are esterified with methyl groups, also demonstrates anticancer and immunostimulating activities *in vivo*. An antitumor activity assay demonstrated that HRWP-A could significantly inhibit Lewis lung carcinoma (LLC) growth in tumor-bearing mice. In addition, this compound enhanced lymphocyte proliferation, augmented macrophage activities, and promoted natural killer cell activity in tumor-bearing mice. The authors used three different doses of polysaccharide (50, 100, and 200 mg/kg), which were administrated intragastrically each day for 14 days.

## Radioprotective ability of sea buckthorn

Due to its high content of biologically-active compounds and antioxidants, sea buckthorn is included in cancer therapy for its radioprotective activity, which has been demonstrated in a number of studies by Goel et al. (2002, 2003a,b, 2004, 2005). Agrawala and Goel (2002) found whole extract of fresh sea buckthorn berries to have protective properties (*H. rhamnoides*—RH-3; 25–35 mg/kg body wt), particularly for radiation-induced micronuclei in mouse bone marrow. In addition, Goel et al. (2002) found that RH-3 inhibited the Fenton reaction and radiation-mediated production of hydroxyl radicals *in vitro*.

Kumar et al. (2002) report that RH-3 inhibited DNA strand breaks induced by radiation and tertiary butyl hydroperoxide in a dose-dependent manner, as revealed by Comet assay. They also note a strong compaction of chromatin occurring at concentrations of 100 and 120 pg/ml RH-3 and above, which made the nuclei resistant to radiation, even at a dose of 1,000 Gy. Goel et al. (2003a) report the protection of jejunal crypts by RH-3 against lethal whole body gamma irradiation (10 Gy), and that caspase-3 activity was also significantly lower in mice administered RH-3 before irradiation as compared to irradiated controls. Interestingly, a radioprotective dose of RH-3 (30 mg/kg b.w.) induced significant DNA fragmentation (studied spectrofluorimetrically) in thymocytes in mice *in vivo*. In addition, sea buckthorn treatment before irradiation was found to enhance radiation-induced apoptosis *in vivo* (Goel et al., 2004). Goel et al. (2005) suggest also that pre-irradiation treatment of mice with 30 mg/kg sea buckthorn berry extract protects the functional integrity of mitochondria from radiation-induced oxidative stress. These experiments examined the levels of various biomarkers of oxidative stress, including superoxide anion, lipid peroxidation, and protein oxidation. Interestingly, RH-3 was found to have immunostimulatory properties, which may play an important role in its radioprotective efficacy (Prakash et al., 2005).

## Conclusion

Although many studies have confirmed the anticancer activity of sea buckthorn, its medicinal and prophylactic doses remain unknown, and no clinical trials have yet been performed: only *in vitro* or *in vivo* studies involving experimental animals. It is known that sea buckthorn may participate in the prevention and treatment of cancer; it also accelerates the return to health of patients receiving chemotherapy by significantly improving the performance of the immune system and relieves hematological damage.

**Figure 1:**
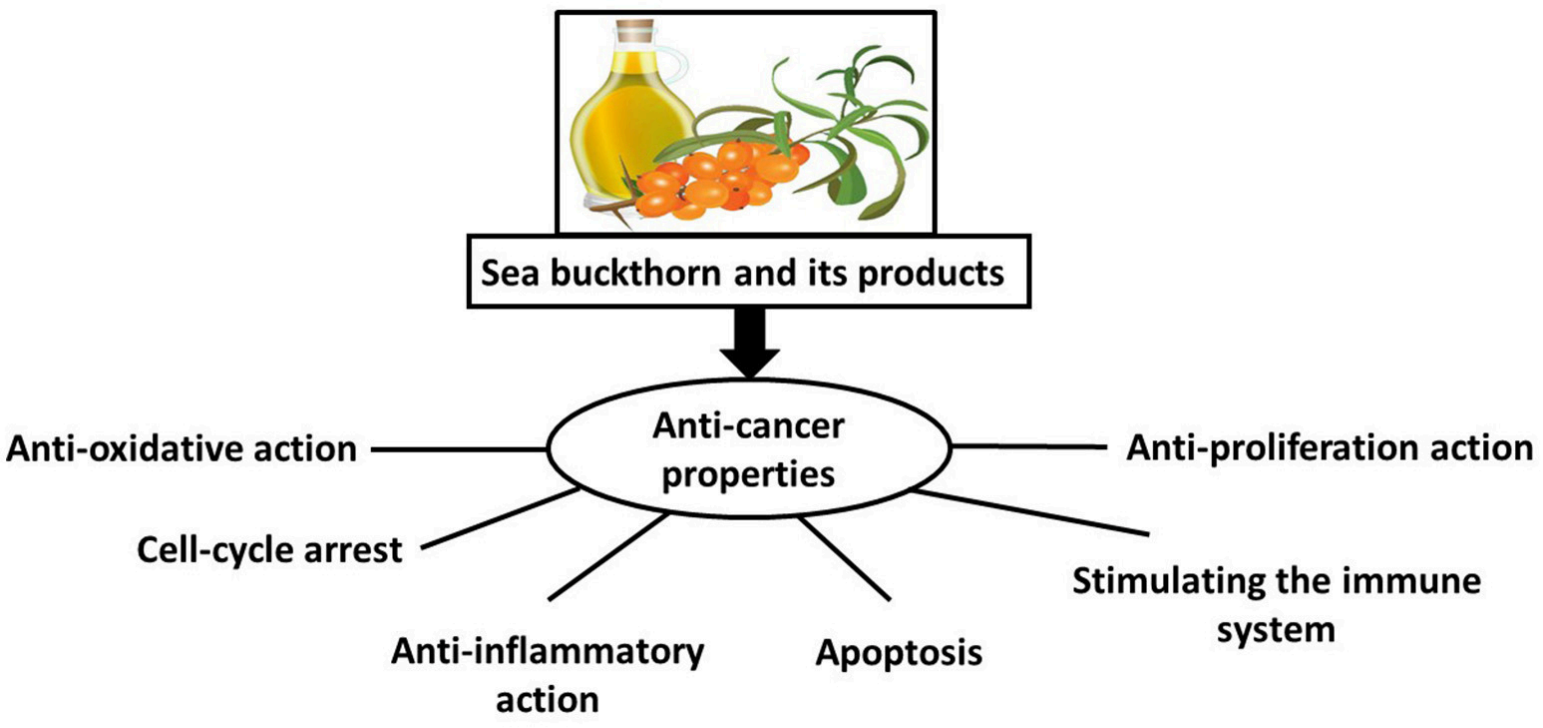
Hypothetical mechanisms of action by which sea buckthorn may evoke chemopreventive and therapeutic responses against cancer.

The hypothetical mechanism by which sea buckthorn may exert its chemopreventive and therapeutic responses against cancer is presented in Figure 1. The bioactive substances in various parts of sea buckthorn have a range of properties, including antioxidant, anti-inflammatory, and anti-proliferative activities; they also induce apoptosis and strengthen the immune system; however, the molecular mechanisms remain unclear. Therefore, before sea buckthorn can be considered the “golden mean” for treatment of cancers, it requires further study in a range of high-quality studies.

## Author contributions

All authors (BO, BS, KU) listed have made a substantial, direct and intellectual contribution to the work, and approved it for publication.

### Conflict of interest statement

The authors declare that the research was conducted in the absence of any commercial or financial relationships that could be construed as a potential conflict of interest. The reviewer BW and handling Editor declared their shared affiliation.

## Abbreviations

Bax: the pro-apoptotic protein Bcl-2-associated X
FAS: fatty acid synthase
GA: gallic acid
HRWP-A: a water-soluble homogenous polysaccharide
IRF-1: interferon regulatory factor-1
LLC: Lewis lung carcinoma
NFκB: nuclear factor kappa-light-chain-enhancer of activated B cells
RH-3: whole extract of fresh sea buckthorn berries
TNF: tumor necrosis factor.
